# Supplementary material for: Physical Fitness, Executive Functions, and Academic Performance in Children and Youth: A Cross-Sectional Study
Source: Behav Sci (Basel). 2024 Nov 1;14(11):1022. doi: 10.3390/bs14111022 (PMC11591446; doi:10.3390/bs14111022)
Supplement: Supplementary file 1 [file behavsci-14-01022-s001.zip › behavsci-3264775-supplementary.pdf]

Supplementary Materials

Physical Fitness, Executive Functions, and Academic Performance in Children and Youth: A Cross-Sectional Study

Valter Fernandes, Arthur Silva, Andrea Carvalho, Sidarta Ribeiro and Andrea Deslandes

Table S1 - Descriptive analysis of the sample categorized according to performance in executive functions.

	Low EF N=65	High EF N=66	p	Total 131	Min - Max
Sex	29 fem. (45%)	30 fem. (58%)		64 fem. (49%)	
Weight (kg)	45.61 (11.62)	45.6 (11.39)	0.92	45.61 (11.46)	25.9 - 87
High (cm)	153.64 (8.69)	152.23 (7.59)	0.42	152.93 (8.15)	134.2 - 173
BMI (score)	19.13 (3.65)	19.48 (4.12)	0.704	19.31 (3.89)	13 - 33
PHV (years)	13.01 (1.19)	13.23 (1)	0.228	13.12 (1.1)	11 - 15.7
Age (years)	12.77 (1.2)	12.38 (0.92)	0.084	12.57 (1.08)	10.8 - 15.9
Agility (s)	8.1 (0.89)	7.51 (0.66)	<0.001*	7.8 (0.84)	5.1 - 10.9
TTD[1] (s)	20.25 (3.15)	18.73 (2.08)	0.001*	19.48 (2.76)	13.9 - 28.1
LLEST (cm)	122.5 (30.77)	130.06 (26.31)	0.085	126.31 (28.75)	45.6 - 280
ULEST (cm)	241.27 (66.49)	252.83 (66.11)	0.108	247.09 (66.29)	70.5 - 450.3
FitC[1]	174.79 (74.96)	227.86 (72.57)	<0.001*	201.53 (78.16)	20.61 - 387.4
Corsi	33.02 (13.17)	36.17 (17.27)	0.309	34.61 (15.4)	2 - 104
Digit	8.02 (1.68)	8.46 (1.66)	0.331	8.24 (1.68)	0 - 13
HFT	12.64 (2.5)	8.67 (1.3)	<0.001*	10.64 (2.81)	4.61 - 26.2
SPT-R	58.87 (6.74)	61.35 (7.07)	0.019	60.12 (6.99)	37 - 72.13
Portuguese	5.55 (1.44)	5.88 (1.6)	0.247	5.71 (1.52)	1.14 - 9.38
Math[1]	6.18 (1.39)	6.83 (1.44)	0.010	6.51 (1.45)	3 - 9.9
Grades	6.38 (0.94)	6.68 (1.08)	0.151	6.53 (1.02)	4.2 - 9.2
AcadC	7.39 (0.58)	7.72 (0.77)	<0.001*	7.56 (0.7)	5.6 - 9.46

Notes: Measures are expressed as mean and standard deviation. Smaller values for s (seconds) correspond to better results. The remaining variables are scores, with higher scores corresponding to better results; P-results for Mann-Whitney test significance (except 1 = T-test significance), after Bonferroni correction = $p<0.003$; BMI (body mass index); PHV (peak high velocity); TTD (touch disc test); LLEST (lower limb explosive strength test); ULEST (upper limb explosive strength test); FitC (fitness composite score); Corsi (total score of the Corsi´s block task); Digits (sum score up to two errors of the forward and backward digit span); HFT (cost of accurate responses of the Hearts and Flowers executive functions task); SPT-R (Reading School Performance Test); Grades (Overall School Grades); AcadC (composite score of academic performance).
